# Renal Biomarkers and Prognosis in HFpEF and HFrEF: The Role of Albuminuria and eGFR—A Systematic Review

**DOI:** 10.3390/medicina61081386

**Published:** 2025-07-30

**Authors:** Claudia Andreea Palcău, Livia Florentina Păduraru, Cătălina Paraschiv, Ioana Ruxandra Poiană, Ana Maria Alexandra Stănescu

**Affiliations:** 1 Faculty of Medicine, “Carol Davila” University of Medicine and Pharmacy, 050474 Bucharest, Romania; 2 Department of Cardiology, Elias University Hospital, 011461 Bucharest, Romania; 3 Department of Family Medicine, “Carol Davila” Central Military Emergency University Hospital, 051075 Bucharest, Romania; 4 Academy of Romanian Scientists (AOSR), 050091 Bucharest, Romania; 5 “Emil Palade” Center of Excellence for Young Researchers, The Academy of Romanian Scientists, 030167 Bucharest, Romania

**Keywords:** heart failure, albuminuria, estimated glomerular filtration rate (eGFR), prognosis, ejection fraction

## Abstract

*Background and Objectives*: Heart failure (HF) and chronic kidney disease (CKD) frequently coexist and are closely interrelated, significantly affecting clinical outcomes. Among CKD-related markers, albuminuria and estimated glomerular filtration rate (eGFR) have emerged as key prognostic indicators in HF. However, their specific predictive value across different HF phenotypes—namely HF with preserved ejection fraction (HFpEF) and HF with reduced ejection fraction (HFrEF)—remains incompletely understood. This systematic review aims to evaluate the prognostic significance of albuminuria and eGFR in patients with HF and to compare their predictive roles in HFpEF versus HFrEF populations. *Materials and Methods*: We conducted a systematic search of major databases to identify clinical studies evaluating the association between albuminuria, eGFR, and adverse outcomes in HF patients. Inclusion criteria encompassed studies reporting on cardiovascular events, all-cause mortality, or HF-related hospitalizations, with subgroup analyses based on ejection fraction. Data extraction and quality assessment were performed independently by two reviewers. *Results*: Twenty-one studies met the inclusion criteria, including diverse HF populations and various biomarker assessment methods. Both albuminuria and reduced eGFR were consistently associated with increased risk of mortality and hospitalization. In HFrEF populations, reduced eGFR demonstrated stronger prognostic associations, whereas albuminuria was predictive across both HF phenotypes. Heterogeneity in study design and outcome definitions limited comparability. *Conclusions*: Albuminuria and eGFR are valuable prognostic biomarkers in HF and may enhance risk stratification and clinical decision-making, particularly when integrated into clinical assessment models. Differential prognostic implications in HFpEF versus HFrEF highlight the need for phenotype-specific approaches. Further research is warranted to validate these findings and clarify their role in guiding personalized therapeutic strategies in HF populations. The current evidence base consists primarily of observational studies with variable methodological quality and inconsistent reporting of effect estimates.

## 1. Introduction

Heart failure (HF) remains a leading global health burden, with rising prevalence despite advances in therapy. Over the past decade, the proportion of patients with heart failure with preserved ejection fraction (HFpEF) has significantly increased, and this phenotype currently lacks specific evidence-based treatment options [1]. At the same time, chronic kidney disease (CKD) is frequently observed in HF populations and substantially worsens outcomes through a bidirectional pathophysiological relationship known as the cardiorenal syndrome. Simple and widely available renal biomarkers, such as albuminuria and estimated glomerular filtration rate (eGFR), reflect not only renal damage and function but also systemic processes, including endothelial dysfunction and inflammation, that contribute to HF development and progression [2]. While these biomarkers are well established in nephrology, their comparative prognostic value in HF, and particularly their ability to distinguish risk across HF phenotypes, has not been systematically synthesized. Addressing this gap may inform more accurate risk stratification and early identification of high-risk individuals.

HF and CKD frequently coexist and mutually influence each other’s progression and clinical outcomes. The shared physiological mechanisms by which the heart and kidneys regulate blood pressure, sodium balance, and water homeostasis may result in dysfunction of one organ due to impairment of the other, highlighting their complex interrelationship [1].

The burden of CKD significantly contributes to the increasing prevalence of HF. CKD is a major public health issue and ranks among the leading causes of death globally. Nearly half of all individuals with CKD remain unaware of their condition, as symptoms often appear only in the advanced stages of the disease [3].

Serum creatinine and albuminuria are well-established markers of CKD and are also important indicators of both the development and progression of HF. Albuminuria is commonly observed in patients with HF; however, its underlying causes and the mechanisms driving disease progression and adverse outcomes remain incompletely understood. Interventions aimed at reducing albuminuria have shown potential in reducing the risk of incident HF or slowing the progression of existing disease [2].

The lifetime risk of developing HF has increased by approximately 24% in recent years. Recent epidemiological data indicate that one in four individuals is at risk of developing HF during their lifetime [4].

HFpEF is defined by typical signs and symptoms of HF, with evidence of structural and/or functional cardiac abnormalities, elevated natriuretic peptide levels, and a left ventricular ejection fraction greater than 50% [5]. Although the overall incidence of HF appears to have plateaued globally and is declining in developed countries [6], its prevalence continues to rise—particularly the prevalence of the HFpEF phenotype, which is increasing more rapidly in men than in women [4]. Elevated urinary albumin-to-creatinine ratio (UACR) is independently associated with worse outcomes in HFpEF [7].

Similarly, CKD is also highly prevalent among patients with HF with reduced ejection fraction (HFrEF) and carries important therapeutic implications, particularly in advanced stages. As one of the most common comorbidities, CKD contributes significantly to the risk of all-cause mortality and HF-related hospitalizations in HFrEF. These therapeutic challenges become more pronounced in advanced CKD stages, with patients in stage 4 or 5 at higher risk of receiving suboptimal guideline-directed therapy [8].

Several studies have suggested that albuminuria may provide prognostic information not only in established HF but also in asymptomatic or high-risk populations, including individuals with hypertension, diabetes, or other cardiovascular risk factors. Early associations between low-grade albuminuria and increased cardiovascular risk have been reported even when albumin excretion is below the conventional threshold for CKD diagnosis. These observations have raised the question of whether albuminuria could serve as an early and accessible biomarker for risk stratification and HF prevention.

Therefore, this systematic review aims to synthesize current evidence to determine whether the prognostic value of renal biomarkers differs between HF phenotypes and to evaluate whether integrating albuminuria and estimated glomerular filtration rate (eGFR) into the risk stratification process can improve the early identification and monitoring of patients at high-risk for adverse outcomes.

## 2. Pathophysiological Background of Albuminuria in HFpEF and HFrEF

The concept of HFpEF has evolved substantially in recent years due to the high number of patients who exhibit signs and symptoms of HF despite having a normal or near-normal ejection fraction (EF). This suggests that the heart’s ability to relax and fill during diastole is impaired. The current challenge lies in understanding the underlying pathophysiological mechanisms and, consequently, in identifying effective treatment strategies for this population.

Albuminuria, defined as the abnormal excretion of albumin in the urine, has emerged as a novel prognostic marker in patients with HFpEF, indicating a potential link between renal dysfunction and the pathogenesis and progression of this HF phenotype [9]. Albuminuria in the presence of a normal glomerular filtration rate (GFR) may be attributed to HF itself, particularly due to elevated central venous pressure, which leads to increased renal venous congestion, reduced renal perfusion pressure, and, ultimately, a decline in GFR [2].

In HFrEF, one of the key mechanisms contributing to disease progression is activation of the renin-angiotensin-aldosterone system (RAAS), resulting in sodium retention and extracellular fluid expansion, which further exacerbates edema and worsens HF [10]. Although neurohormonal activation initially plays a compensatory role in impaired cardiac function, it has deleterious long-term effects. Sympathetic nervous system overactivation leads to a reduction in EF, fluid overload, and further stimulation of the RAAS pathway [11]. RAAS activation, in turn, increases glomerular hydrostatic pressure, thereby contributing to the development of albuminuria [12].

An important aspect to consider is the presence of diabetes mellitus (DM) and its implications for renal function in patients with HF. DM is a well-established contributor to both microvascular and macrovascular complications, and its impact on the cardiorenal axis is particularly significant. Chronic hyperglycemia promotes glomerular endothelial dysfunction, thickening of the basement membrane, and mesangial expansion, leading to increased glomerular permeability and the development of albuminuria [13]. In patients with HF, especially those with HFpEF, DM amplifies systemic inflammation, oxidative stress, and myocardial stiffness, further impairing diastolic function [14]. In HFrEF, the coexistence of diabetes is associated with a more rapid decline in renal function and higher levels of neurohormonal activation [15]. Notably, albuminuria is more prevalent and carries greater prognostic significance in diabetic patients, correlating strongly with adverse cardiovascular outcomes and with elevated levels of NT-proBNP, which may reflect combined hemodynamic and metabolic stress. Therefore, the presence of DM should be considered when interpreting albuminuria and stratifying risk in patients with HF [16].

**Figure 1:**
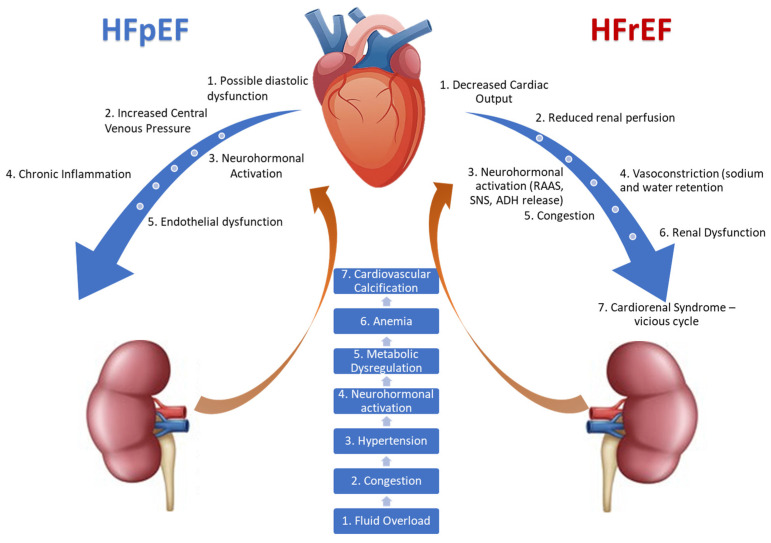
The shared pathophysiological mechanisms underlying the cardiorenal axis.

The complex interrelationship between HFpEF, HFrEF, and renal function is illustrated in Figure 1.

## 3. Materials and Methods

This systematic review was conducted in accordance with the PRISMA 2020 guidelines for reporting systematic reviews [17], as illustrated in Figure 1. A review protocol was not registered in a public database; however, all methodological steps were predefined and consistently applied to ensure transparency and reproducibility.

### 3.1. Search Strategy and Information Sources

The literature search was performed using three electronic databases: PubMed, Web of Science, and Scopus, which covered articles published between January 2014 and December 2024, selected to ensure alignment with recent diagnostic criteria for HF and with standardized reporting of albuminuria and eGFR. The last search was conducted on 10 January 2025. Search strategies combined MeSH terms and keywords for “heart failure”, “HFpEF”, “HFrEF”, “albuminuria”, “urinary albumin-to-creatinine ratio”, “eGFR,” and “renal biomarkers”, using Boolean operators. Detailed search strings for each database are provided in the Supplementary Material. Reference lists of all included articles were manually reviewed to identify additional eligible studies.

The search strategy was developed based on a structured PICO framework, as follows:

Population (P): patients diagnosed with HF.

Intervention (I): assessment of albuminuria and estimated glomerular filtration rate.

Comparators (C): patients with HFpEF compared to those with HFrEF.

Outcomes (O): prognostic significance and predictive value of albuminuria and eGFR regarding cardiovascular events, mortality, and long-term clinical outcomes.

### 3.2. Eligibility Criteria

The inclusion and exclusion criteria applied in the study selection process are summarized in Table 1. These criteria were defined to identify studies evaluating the prognostic value of albuminuria and/or eGFR in adult patients with HF, stratified by ejection fraction phenotype. Only peer-reviewed, English-language original research articles were considered.

We included prospective, retrospective, case-control, and cross-sectional studies, as well as registry analyses of adults (>18 years) at high cardiovascular risk or with diagnosed HF (HFpEF, HFmrEF, or HFrEF). Eligible studies reported clearly defined measurements of albuminuria (UACR or dipstick) and/or eGFR and clinical outcomes, including cardiovascular events, mortality, HF-related hospitalizations, or long-term outcomes. Studies were excluded if they (1) involved pediatric or pregnant populations, (2) were reviews, commentaries, or editorials, (3) lacked data on biomarker assessment or relevant outcomes, or (4) presented major methodological flaws or a high-risk of bias.

### 3.3. Study Selection

**Figure 2:**
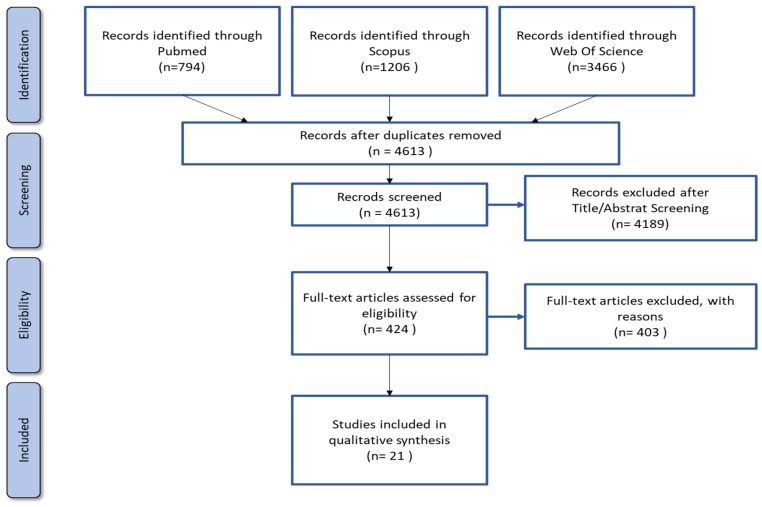
The preferred reporting items for systematic reviews and meta-analyses (PRISMA) flow chart.

We used EPPI-Reviewer, a web-based software platform, for the management of records, study selection, and data extraction [18]. Two independent reviewers screened titles and abstracts, assessed full texts for eligibility, and resolved discrepancies through discussion or by consulting a third reviewer. A total of 5466 records were initially identified, of which 4613 remained after duplicate removal. After screening, 424 full text articles were assessed for eligibility, and 21 studies met the inclusion criteria and were included in this review. These numbers are illustrated in the PRISMA flow diagram (Figure 2).

### 3.4. Data Extraction and Synthesis

For each included study, data were extracted on study design, country, sample size, patient characteristics (age, sex, comorbidities), HF phenotype (HFpEF, HFrEF, HFmrEF), biomarker type and measurement methods, outcome definitions, and follow-up duration.

Due to heterogeneity in study designs, biomarker definitions, and outcomes, a meta-analysis was not feasible. Results were synthesized narratively and summarized in structured tables (Table 2, Table 3 and Table 4). Studies were grouped by HF phenotype, biomarker type, and outcome category.

### 3.5. Risk of Bias and Certainty Assessment

The certainty of the evidence for each outcome was evaluated using the GRADE (Grading of Recommendations, Assessment, Development and Evaluation) [19] approach. A detailed summary is provided in Supplementary Materials Table S6. The risk of bias was assessed using the Newcastle–Ottawa Scale (NOS) [20], which evaluates the quality of observational studies across three domains: selection, comparability, and outcome/exposure assessment. Two independent reviewers assessed the risk of bias, resolving any disagreements through discussion. The results of the NOS evaluation are presented in Supplementary Materials Table S5.

## 4. Results

This systematic review included a total of 21 studies, published between 2014 and 2024, investigating the prognostic significance of albuminuria and estimated glomerular filtration rate (eGFR) in relation to HF phenotypes and cardiovascular risk. The included studies were conducted in a wide range of geographical settings—including Europe (Sweden, Germany, UK), North America (USA, Canada), and Asia (China, South Korea, Thailand)—allowing for more diverse clinical implications.

Study populations varied considerably, ranging from asymptomatic individuals at high cardiovascular risk or enrolled in screening programs to patients with established diagnoses of hypertension, type 2 diabetes mellitus (T2DM), CKD, or overt HF (HFpEF, HFmrEF, and HFrEF). Most studies were prospective or retrospective observational in design and used UACR as the primary measure of albuminuria.

Given the strong pathophysiological link between heart and kidney function—particularly through mechanisms such as neurohormonal activation, volume overload, and microvascular dysfunction—the findings of these studies provide valuable insights into the role of renal biomarkers in risk stratification. The results are summarized below and structured into three main thematic areas: the prognostic value of albuminuria in high-risk or asymptomatic populations, its association with cardiac remodeling and dysfunction, and its implications across different heart failure phenotypes.

### 4.1. Prognostic Value of Urinary Albumin-to-Creatinine Ratio in Asymptomatic Individuals and High-Risk Populations

Five studies, with sample sizes ranging from 635 to 198,637 participants, evaluated the prognostic relevance of albuminuria in asymptomatic individuals and high-risk populations without diagnosed HF. These studies consistently reported an association between urinary albumin excretion—even at levels below the current upper limit of normal—and cardiovascular outcomes.

The findings of the included studies are summarized in Table 2, focusing strictly on the measured outcomes and reported associations, without interpretation. These data illustrate the potential role of albuminuria as an early marker of cardiovascular risk in various high-risk populations.

**Table 2:** Key clinical studies assessing the association between UACR and cardiovascular risk in non-HF populations. Abbreviations: UACR = urinary albumin-to-creatinine ratio; MACE = major adverse cardiovascular events; HF = heart failure; CV = cardiovascular; HTN = hypertension; CVD = cardiovascular disease; eGFR = estimated glomerular filtration rate.

Author	Study Type and Inclusion Criteria	Patients Number	Evaluated Parameters	Measured Outcome	Key Findings
LIU (2018) [21]	Retrospective (patients with high CV risk)	1474	UACR (quantitative)	MACE, all-cause mortality, HF hospitalization	UACR > 30 mg/g was associated with a twofold increase in all-cause mortality and a threefold increase in HF hospitalization.
AIUMTRAKUL (2021) [22]	Retrospective (cohort study—rural Thai population)	635	Urine albumin dipstick (semi-quantitative)	CV and renal complications	Macroalbuminuria independently predicted poor CV and renal outcomes.
ARNLOV and NOWAK (2022) [23]	Prospective (patients without hypertension, diabetes, or CVD)	198,637	UACR	CV events and all-cause mortality	Albuminuria was strongly associated with a 10-year risk of composite CV outcomes, independent of traditional risk factors.
SUNG (2016) [24]	Prospective	9102	UACR	Incident HTN and CV mortality	Microalbuminuria was associated with increased risk of incident HTN and CV mortality, but not with incident diabetes.
SEO (2017) [25]	Prospective patients from a health screening program (Korea)	32,653	UACR	All-cause mortality and CVD	Low-grade albuminuria (UACR > 5.42 mg/g) was more strongly associated with CVD and all-cause mortality than with HTN, independent of eGFR.

With regard to mortality outcomes, two studies [21,25] found that increased UACR was associated with higher all-cause mortality. Liu et al. reported that older adults (aged over 65) with elevated cardiovascular risk and an ACR greater than 30 mg/g had a twofold increase in risk of all-cause mortality and HF hospitalization [21]. Similarly, the prospective study conducted by Seo et al. [25] assessing the effects of albuminuria and hypertension on all-cause and cardiovascular mortality found that both conditions were independent risk factors. Albuminuria demonstrated a stronger statistical association with cardiovascular outcomes compared to hypertension in this cohort.

For cardiovascular events, Arnlov and Nowak [23] demonstrated in a large prospective cohort of over 198,000 individuals without hypertension, diabetes, or known CVD that microalbuminuria was present in more than 5% of cases and albuminuria was strongly associated with a significantly increased 10-year risk of composite cardiovascular outcomes. The association remained robust after adjusting for conventional risk factors.

In terms of HF and hospitalization, Liu et al. [21] found that participants with elevated UACR had a threefold increased risk of HF hospitalization. Furthermore, in a retrospective analysis, Aiumtrakul et al. [22] demonstrated that even a semi-quantitative assessment of albuminuria predicted adverse cardiovascular and renal outcomes.

Finally, regarding hypertension incidence, Sung et al. [24] showed that microalbuminuria was associated with a higher risk of developing hypertension and cardiovascular mortality, but not with incident diabetes, during follow-up.

### 4.2. Albuminuria as a Marker of Cardiac Remodeling and Dysfunction

Nine studies, with sample sizes ranging from 60 to 4063 participants, investigated the association between urinary albumin excretion and structural or functional cardiac abnormalities, primarily using echocardiographic parameters and cardiac MRI. Table 3 summarizes the characteristics and findings of these studies.

With regard to left ventricular mass, multiple studies [26,27,28] reported that higher UACR levels were associated with increased left ventricular mass index (LVMI). Feng et al. [26] showed that both low eGFR and elevated baseline UACR predicted higher LVMI at follow-up in hypertensive patients.

For diastolic dysfunction, Hanna et al. [27], Wang et al. [28], and Jorgensen et al. [29] found significant correlations between UACR levels and markers of impaired diastolic function, including in patients without overt cardiovascular disease or with only mild albuminuria. On the other hand, in a prospective cohort of 825 patients with non-dialysis CKD, Landler et al. [30] found no independent association between albuminuria and cardiac function, although a borderline relationship with diastolic dysfunction was observed.

In terms of systolic function, Liu et al. [31] reported that elevated UACR was independently associated with reduced global longitudinal strain (GLS), indicating subclinical systolic dysfunction. Abdel-Latif et al. [32] also found an inverse correlation between UACR and LV GLS in normotensive patients with T2DM. Similarly, a retrospective study in 251 patients with T2DM by Patro et al. [33] reported that the severity of albuminuria correlated with parameters of both LV systolic and diastolic function.

Additionally, one study using cardiac MRI [34] identified an association between higher UACR and smaller right ventricular volumes, which were linked to increased all-cause mortality during follow-up.

**Table 3:** Association Between albuminuria (UACR) and cardiac structural changes assessed by imaging modalities. Abbreviations: UACR = urinary albumin-to-creatinine ratio; eGFR = estimated glomerular filtration rate; LV = left ventricle; LVMI = left ventricular mass index; GLS = global longitudinal strain; LVH = left ventricular hypertrophy; RV = right ventricle; T2DM = type 2 diabetes mellitus; HbA1c = glycated hemoglobin; CKD = chronic kidney disease; CV = cardiovascular; *HCHS/SOL = Hispanic Community Health Study/Study of Latinos.

Author	Study Type and Inclusion Criteria	Patient Number	Evaluated Parameters	Measured Outcome	Key Findings
HANNA (2017) [27]	Cross-sectional (patients > 45 years, *HCHS/SOL cohort)	1815	Echocardiography; UACR	LV mass, diastolic function	Higher UACR was associated with greater LV mass and diastolic dysfunction.
FENG (2017) [26]	Prospective (patients with hypertension)	539	Echocardiography; UACR; Serum Creatinine	UACR, eGFR, LVMI	Low eGFR and higher baseline UACR were associated with increased LVMI at follow-up.
JORGENSEN (2018) [29]	Cross-sectional (patients with T2DM)	915	Echocardiography; UACR	Diastolic and systolic function	Microalbuminuria was associated with diastolic dysfunction; macroalbuminuria with reduced systolic function.
LIU (2020) [31]	Cross-sectional (patients with hypertension and albuminuria)	2857	Echocardiography; UACR	UACR, GLS	Higher UACR was independently associated with impaired subclinical systolic function (lower GLS).
ABDELLATIF (2021) [32]	Prospective (normotensive patients with T2DM)	60	Echocardiography; UACR; HbA1c	GLS	Albuminuria was associated with lower average LV GLS.
SYED (2023) [34]	Prospective (patients without clinical CV disease)	4063	Cardiac MRI; eGFR; UACR	RV volumes; all-cause mortality	Higher albuminuria and smaller RV volumes were associated with increased mortality risk, independent of eGFR and LV parameters.
LANDLER (2022) [30]	Prospective (patients with non-dialysis CKD)	825	Echocardiography; eGFR; UACR	Cardiac function parameters	No independent association was found between albuminuria and cardiac function; a borderline association existed with diastolic dysfunction.
WANG (2019) [28]	Cross-sectional	870	Echocardiography; UACR	LVH; LV diastolic function	Low-grade albuminuria was associated with LVH and diastolic dysfunction, even in patients with normal UACR levels.
PATRO (2021) [33]	Retrospective (patients with T2DM)	251	Echocardiography; UACR	LV systolic and diastolic function	The severity of albuminuria was significantly correlated with parameters of LV systolic and diastolic function.

### 4.3. Prognostic Implications of Albuminuria (UACR) Across Heart Failure Phenotypes: HFpEF and HFrEF

Seven studies, with sample sizes ranging from 144 to 9287 participants, evaluated the prognostic implications of albuminuria and eGFR in patients with different HF phenotypes (Table 4).

In a prospective study of 144 patients with HFpEF, Katz et al. [7] reported that a higher UACR was associated with increased LV mass index, impaired GLS, and RV remodeling and was an independent prognostic marker for worse outcomes.

At diagnosis, Boorsma et al. [35] showed that not all patients with HF present with micro- or macroalbuminuria. They reported that approximately 10% of patients had macroalbuminuria (UACR > 300 mg/g), while nearly 35% had microalbuminuria (UACR between 30 and 300 mg/g).

Gori et al. [36], analyzing data from the PARAMOUNT trial, found that renal dysfunction was common in HFpEF and was associated with cardiac remodeling and systolic function parameters. However, in that cohort, albuminuria alone did not reach statistical significance in univariate analysis.

In a large cohort of hospitalized patients with acute decompensated heart failure (ADHF), Wang et al. [37] found that higher UACR predicted adverse clinical outcomes, especially in individuals with LVEF ≥ 40%.

Another prospective study of approximately 9000 patients with T2DM and no history of HF demonstrated that elevated UACR levels were independently associated with the incidence of new-onset HF, including values within the normal range [38].

Sharma et al. [39], in a post hoc analysis of patients with T2DM and established cardiovascular disease, reported that UACR was associated with increased risk of incident kidney events, cardiovascular death, heart failure hospitalization (HHF), and all-cause mortality.

Alataş et al. [40] evaluated 426 patients with acute HF and found that microalbuminuria predicted in-hospital mortality in patients with HFrEF and HFmrEF, but not in those with HFpEF.

**Table 4:** UACR and its association with cardiac structure, function, and outcomes in heart failure phenotypes. Abbreviations: UACR = urinary albumin-to-creatinine ratio; HF = heart failure; HFpEF = heart failure with preserved ejection fraction; HFrEF = heart failure with reduced ejection fraction; HFmrEF = heart failure with mildly reduced ejection fraction; ADHF = acute decompensated heart failure; HHF = hospitalization for heart failure; CV = cardiovascular; LVMI = left ventricular mass index; GLS = global longitudinal strain; LV = left ventricle; RV = right ventricle; eGFR = estimated glomerular filtration rate; LVEF = left ventricular ejection fraction; T2DM = type 2 diabetes mellitus; CVD = cardiovascular disease.

Author	Study Type and Inclusion Criteria	Patient Number	Evaluated Parameters	Measured Outcome	Key Findings
KATZ (2014) [7]	Prospective (patients with HFpEF)	144	Echocardiography; UACR	LVMI, GLS, RV remodeling	High UACR was a prognostic marker in HFpEF, independently associated with worse clinical outcomes.
GORI (2014) [36]	Cross-sectional (HFpEF patients from the PARAMOUNT study)	217	Echocardiography; eGFR; UACR	LVMI, LV wall thickness, LV geometry	UACR was associated with increased LVMI and abnormal LV geometry in patients with HFpEF.
BOORSMA (2023) [35]	Retrospective post hoc analysis (HFpEF and HFrEF)	1431	UACR; HF status	Congestive status; mortality; HF (re)hospitalization	Albuminuria was associated with congestive status and increased risk of mortality and HF (re)hospitalization.
ALATAȘ (2022) [40]	Retrospective (patients with AHF: HFpEF, HFmrEF, HFrEF)	426	Microalbuminuria	In-hospital mortality	Microalbuminuria predicted in-hospital mortality in HFrEF and HFmrEF but not in HFpEF.
WANG (2021) [37]	Retrospective (hospitalized patients with ADHF)	1818	UACR	All-cause mortality; CV mortality; HF hospitalization; MACE	High UACR predicted adverse clinical outcomes in patients with ADHF, especially those with LVEF ≥ 40%.
TAO (2023) [38]	Prospective (T2DM patients without HF)	9287	UACR	New-onset HF	Elevated UACR, even within the normal range, was an independent risk factor for incident HF in T2DM patients.
SHARMA (2023) [39]	Post hoc analysis (T2DM patients with CVD)	7020	UACR	Kidney events; CV death; HF hospitalization; all-cause mortality	Albuminuria was associated with increased risk of kidney events, CV death, HF hospitalization, and mortality.

## 5. Discussion

This systematic review synthesized evidence on the prognostic value of albuminuria and eGFR in patients across the HF spectrum. The analysis revealed consistent associations between elevated UACR and adverse clinical outcomes, including mortality, cardiovascular events, structural cardiac changes, and HF hospitalization. These associations varied depending on the HF phenotype, with a more robust prognostic value observed in patients with HFrEF and HFmrEF compared to HFpEF.

Both eGFR and albuminuria have been established as significant prognostic biomarkers in HF, although their predictive value may differ between HFrEF and HFpEF phenotypes. Several studies suggest that reduced eGFR is a strong predictor of adverse outcomes in HFrEF, whereas its prognostic accuracy may be less robust in HFpEF. Nonetheless, the incidence of HFpEF is higher in individuals with CKD, suggesting a complex and distinct pathophysiological relationship that warrants phenotype-specific evaluation strategies.

Currently, albuminuria is primarily used to stage CKD, but it is not yet routinely incorporated into cardiovascular risk stratification, despite being recognized as a surrogate marker of CVD [41]. Baseline and longitudinal screening for both eGFR and UACR may improve risk prediction and allow for earlier identification of subclinical cardiac involvement [26].

Several studies suggest that albuminuria may serve as an early and accessible biomarker for cardiovascular risk assessment. Notably, in a large prospective cohort, albuminuria was identified as an early indicator of cardiovascular risk even in the absence of traditional factors such as hypertension or diabetes. Moreover, its inclusion in established risk prediction models improved overall prognostic accuracy beyond that of eGFR alone [23].

Emerging evidence indicates that albuminuria, even below the diagnostic threshold for CKD, is associated with an increased risk of cardiovascular events [42,43] This highlights the potential of UACR as an early marker of cardiovascular dysfunction in patients without overt renal disease. However, the cost-effectiveness of universal albuminuria screening—particularly in non-diabetic, non-hypertensive individuals—remains insufficiently investigated. Preliminary data suggest that targeted screening in individuals over the age of 50 may be both clinically effective and economically feasible [23]. Current guidelines recommend UACR testing every six months in patients with diabetes and every five years in those with hypertension, but optimal screening frequency for other populations has yet to be established [2].

Structural cardiac abnormalities tend to become clinically apparent in more advanced stages of HF, when the cardiovascular consequences of albuminuria are more pronounced. Albuminuria has been linked to endothelial dysfunction and microvascular damage, mechanisms that are thought to underlie left ventricular (LV) remodeling, hypertrophy, and both systolic and diastolic dysfunction [26,27,44].

Elevated UACR levels have consistently been associated with LVMI, impaired GLS, and altered diastolic function parameters. These findings support the utility of albuminuria as a marker of subclinical myocardial injury, particularly in individuals with type 2 DM, hypertension, or other cardiovascular risk factors [7].

The relationship between HF, CKD, and pulmonary hypertension (PH) has also been investigated. CKD is a known risk factor for PH, with a reported prevalence of nearly 35% in this population. The association between CKD and HF is bidirectional and may involve overlapping mechanisms such as inflammation, pressure and volume overload, and endothelial dysfunction. Among the etiologies of PH, HFpEF is frequently underrecognized [39], despite multiple studies showing that PH and CKD independently predict mortality in HF populations [45,46,47,48]. This association may be partially mediated by chronic diastolic dysfunction and volume expansion, although further research is needed to clarify the underlying mechanisms.

Incorporating UACR into cardiovascular risk models may significantly enhance risk stratification in patients with T2DM, particularly for identifying early stages of HF. In a prospective study of approximately 9000 individuals with T2DM and no history of HF, Tao et al. [38] demonstrated that elevated UACR was independently associated with the development of new-onset HF.

### Unanswered Questions and Future Directions

Despite growing evidence supporting the prognostic value of albuminuria and eGFR in HF, several critical questions remain unresolved. The exact pathophysiological mechanisms linking albuminuria to HFpEF progression are not fully elucidated and may involve a combination of systemic endothelial dysfunction, renal venous congestion, and microvascular injury. Further mechanistic studies are needed to clarify these interactions.

The prognostic value of albuminuria appears to vary across HF phenotypes, yet the reasons behind these differences remain unclear. Variability in HFpEF definitions and albuminuria measurement methods may partly account for inconsistent findings and limit cross-study comparability. Standardization of both biomarker assessment and HF classification will be essential for future studies.

Although UACR screening is well established in patients with DM or hypertension, the optimal frequency and cost-effectiveness of testing in lower-risk populations—such as non-diabetic, non-hypertensive adults—remain to be determined. Large-scale prospective studies are required to address these gaps. Future studies should also investigate the combined use of UACR with other biomarkers (e.g., natriuretic peptides, troponins) in multiparametric risk models for HF.

Finally, while therapies such as RAAS inhibitors and SGLT2 inhibitors have been shown to reduce albuminuria, it is not yet known whether targeting albuminuria directly improves HF outcomes. Dedicated trials are needed to explore the role of albuminuria as a modifiable treatment target, particularly in HFpEF.

## 6. Strengths and Limitations

This systematic review has several strengths. It includes a broad range of studies from multiple geographic regions and diverse patient populations, covering both asymptomatic individuals and patients with diagnosed HFpEF or HFrEF. The structured synthesis of findings across key clinical outcomes—such as mortality, HF hospitalization, and cardiac remodeling—provides a comprehensive overview of the prognostic relevance of albuminuria and eGFR in heart failure.

However, several limitations must be acknowledged. First, the majority of included studies were observational, which limits the ability to infer causality. Second, heterogeneity in study design, definitions of albuminuria, measurement methods, and patient populations may affect the general applicability of the findings. Third, although efforts were made to standardize the extraction and reporting of outcomes, differences in statistical adjustment and endpoint definitions across studies may introduce bias or limit direct comparisons.

## 7. Conclusions

Albuminuria is a valuable, yet underutilized, prognostic marker in heart failure, with particularly strong relevance for risk stratification in HFpEF. Its presence is independently associated with adverse outcomes, even at levels below the diagnostic threshold for chronic kidney disease. Incorporating albuminuria assessment into routine clinical practice may enable earlier identification of patients at increased risk for heart failure progression and related cardiovascular events.

However, the widespread implementation of albuminuria as a screening tool requires further validation through large-scale prospective studies. Future research should focus on defining the optimal screening intervals, identifying the most appropriate target populations, and evaluating the cost-effectiveness of albuminuria-based strategies within comprehensive cardiovascular prevention and personalized HF management frameworks.

## Figures and Tables

**Table 1:** Inclusion and exclusion criteria. * Based on the latest criteria from the American College of Cardiology/American Heart Association (ACC/AHA) or the European Society of Cardiology (ESC).

Type	Inclusion	Exclusion
Study Design	Prospective, retrospective, case-control studies, cross-sectional studies, and registry analyses	Editorials, letters, commentaries, and narrative reviews
Population	>18 years old; Patients with high cardiovascular (CV) risk or diagnosed with HFpEF/HFrEF *	Pediatric populations; Pregnant women
Outcome and Measurement	Clearly defined measurements of albuminuria and/or eGFR	Studies lacking essential information on measurement protocols; Studies that did not focus on the association between HF and renal outcomes.
Timeframe	2014–2024	Outside the specified timeframe
Language	Studies published in English or with full text available in English	
Quality		Significant methodological flaws or high-risk of bias

## Data Availability

All data generated or analyzed during this study are included in this article. Further inquiries can be directed to the corresponding author.

## Supplementary Materials

The following supporting information can be downloaded at: https://www.mdpi.com/article/10.3390/medicina61081386/s1. Supplementary Materials include the detailed review objectives and rationale, full search strategies for all databases, and summary tables presenting the risk of bias assessment (NOS) and certainty of evidence (GRADE) for the included studies.

## Abbreviations

ACR	albumin-to-creatinine ratio
ADHF	acute decompensated heart failure
CKD	chronic kidney disease
CVD	cardiovascular disease
CV	cardiovascular
DM	diabetes mellitus
eGFR	estimated glomerular filtration rate
EF	ejection fraction
GLS	global longitudinal strain
GRADE	Grading of Recommendations, Assessment, Development and Evaluation
HbA1c	glycated hemoglobin
HF	heart failure
HFmrEF	heart failure with mildly reduced ejection fraction
HFpEF	heart failure with preserved ejection fraction
HFrEF	heart failure with reduced ejection fraction
HHF	hospitalization for heart failure
HTN	hypertension
LVEF	left ventricular ejection fraction
LV	left ventricle
LVH	left ventricular hypertrophy
LVMI	left ventricular mass index
MACE	major adverse cardiovascular events
MRI	magnetic resonance imaging
NOS	Newcastle–Ottawa Scale
PH	pulmonary hypertension
PICO	Population, Intervention, Comparison, Outcome
PRISMA	Preferred Reporting Items for Systematic Reviews and Meta-Analyses
RAAS	renin–angiotensin–aldosterone system
RV	right ventricle
T2DM	type 2 diabetes mellitus
UACR	urinary albumin-to-creatinine ratio

## References

[1] Parmar S.S., Muthuppalaniappan V., Banerjee D. (2023). Gaps in Modern Heart Failure and Chronic Kidney Disease Research. Eur. Cardiol..

[2] Khan M.S., Shahid I., Anker S.D., Fonarow G.C., Fudim M., Hall M.E., Hernandez A., Morris A.A., Shafi T., Weir M.R. (2023). Albuminuria and Heart Failure: JACC State-of-the-Art Review. J. Am. Coll. Cardiol..

[3] Ahmed J., Azhar S., Haq N.U., Hussain S., Stájer A., Urbán E., Gajdács M., Jamshed S. (2022). Awareness of Chronic Kidney Disease, Medication, and Laboratory Investigation among Nephrology and Urology Patients of Quetta, Pakistan. Int. J. Environ. Res. Public Health.

[4] Ahmad T., Alexander K.M., Baker W.L., Bosak K. (2023). Heart Failure Epidemiology and Outcomes Statistics: A Report of the Heart Failure Society of America. J. Card. Fail..

[5] Kittleson M.M., Panjrath G.S., Amancherla K., Davis L.L., Deswal A., Dixon D.L., Januzzi J.L., Yancy C.W. (2023). 2023 ACC Expert Consensus Decision Pathway on Management of Heart Failure With Preserved Ejection Fraction. J. Am. Coll. Cardiol..

[6] Shahim B., Kapelios C.J., Savarese G. (2023). Global Public Health Burden of Heart Failure: An Updated Review. Card. Fail. Rev..

[7] Katz D.H., Burns J.A., Aguilar F.G., Beussink L., Shah S.J. (2014). Albuminuria is independently associated with cardiac remodeling, abnormal right and left ventricular function, and worse outcomes in heart failure with preserved ejection fraction. JACC Heart Fail..

[8] Beldhuis I.E., Lam C.S., Testani J.M., Voors A.A., Van Spall H.G., ter Maaten J.M., Damman K. (2022). Evidence-Based Medical Therapy in Patients with Heart Failure with Reduced Ejection Fraction and Chronic Kidney Disease. Circulation.

[9] Marra A.M., Bencivenga L., D’Assante R., Rengo G., Cittadini A. (2022). Heart failure with preserved ejection fraction: Squaring the circle between comorbidities and cardiovascular abnormalities. Eur. J. Intern. Med..

[10] Sullivan R.D., Mehta R.M., Tripathi R., Reed G.L., Gladysheva I.P. (2019). Renin activity in heart failure with reduced systolic function—New insights. Int. J. Mol. Sci..

[11] Borovac J.A., D’AMario D., Bozic J., Glavas D. (2020). Sympathetic nervous system activation and heart failure: Current state of evidence and the pathophysiology in the light of novel biomarkers. World J. Cardiol..

[12] Min K.D., Matsumoto Y., Asakura M., Ishihara M. (2024). Rediscovery of the implication of albuminuria in heart failure: Emerging classic index for cardiorenal interaction. ESC Heart Fail..

[13] ElSayed N.A., Aleppo G., Bannuru R.R., Bruemmer D., Collins B.S., Ekhlaspour L., Gaglia J.L., Hilliard M.E., Johnson E.L., American Diabetes Association Professional Practice Committee (2023). 2. Diagnosis and Classification of Diabetes: Standards of Care in Diabetes—2024. Diabetes Care.

[14] Paulus W.J., Tschöpe C. (2013). A novel paradigm for heart failure with preserved ejection fraction: Comorbidities drive myocardial dysfunction and remodeling through coronary microvascular endothelial inflammation. J. Am. Coll. Cardiol..

[15] Maack C., Lehrke M., Backs J., Heinzel F.R., Hulot J.-S., Marx N., Paulus W.J., Rossignol P., Taegtmeyer H., Bauersachs J. (2018). Heart failure and diabetes: Metabolic alterations and therapeutic interventions: A state-of-The-Art review from the Translational Research Committee of the Heart Failure Association-European Society of Cardiology. Eur. Heart J..

[16] de Boer I.H., Bangalore S., Benetos A., Davis A.M., Michos E.D., Muntner P., Rossing P., Zoungas S., Bakris G. (2017). Diabetes and hypertension: A position statement by the American diabetes association. Diabetes Care.

[17] Page M.J., McKenzie J.E., Bossuyt P.M., Boutron I., Hoffmann T.C., Mulrow C.D., Shamseer L., Tetzlaff J.M., Akl E.A., Brennan S.E. (2021). The PRISMA 2020 statement: An updated guideline for reporting systematic reviews. Syst. Rev..

[18] Thomas J., Graziosi S., Brunton J., Ghouze Z., O’Driscoll P., Bond M., Koryakina A. (2023). EPPI-Reviewer: Advanced Software for Systematic Reviews, Maps and Evidence Synthesis.

[19] Guyatt G.H., Oxman A.D., Vist G.E., Kunz R., Falck-Ytter Y., Alonso-Coello P., Schünemann H.J. (2008). GRADE: An emerging consensus on rating quality of evidence and strength of recommendations. BMJ.

[20] Wells G.A., Shea B., O’Connell D., Peterson J., Welch V., Losos M., Tugwell P. The Newcastle–Ottawa Scale (NOS) for Assessing the Quality of Nonrandomised Studies in Meta-Analyses. https://www.ohri.ca/programs/clinical_epidemiology/oxford.asp.

[21] Liu M., Liang Y., Zhu J., Yang Y., Ma W., Zhang G. (2018). Albumin-to-creatinine ratio as a predictor of all-cause mortality and hospitalization of congestive heart failure in Chinese elder hypertensive patients with high cardiovascular risks. Clin. Hypertens..

[22] Aiumtrakul N., Phichedwanichskul K., Saravutthikul S., Ottasat K., Visuthitepkul K., Jaruthiti T., Jinawong S., Chanthowong K., Pengsritong V., Horadee N. (2021). Urine albumin dipstick independently predicts cardiovascular and renal outcomes among rural Thai population: A 14-year retrospective cohort study. BMC Nephrol..

[23] Ärnlöv J., Nowak C. (2022). Association between albuminuria, incident cardiovascular events, and mortality in persons without hypertension, diabetes, and cardiovascular disease. Eur. J. Prev. Cardiol..

[24] Sung K.-C., Ryu S., Lee J.-Y., Lee S.H., Cheong E., Hyun Y.-Y., Lee K.-B., Kim H., Byrne C.D. (2016). Urine Albumin/Creatinine Ratio Below 30 mg/g is a Predictor of Incident Hypertension and Cardiovascular Mortality. J. Am. Heart Assoc..

[25] Seo M.H., Lee J.Y., Ryu S., Won Y.S., Sung K.C. (2017). The effects of urinary albumin and hypertension on all-cause and cardiovascular disease mortality in Korea. Am. J. Hypertens..

[26] Feng L., Khan A.H., Jehan I., Allen J., Jafar T.H. (2017). Albuminuria and kidney function as prognostic marker of left ventricular mass among South Asians with hypertension. J. Am. Soc. Hypertens..

[27] Hanna D.B., Xu S., Melamed M.L., Gonzalez F., Allison M.A., Bilsker M.S., Hurwitz B.E., Kansal M.M., Schneiderman N., Shah S.J. (2017). Association of Albuminuria With Cardiac Dysfunction in US Hispanics/Latinos. Am. J. Cardiol..

[28] Wang T., Zhong H., Lian G., Cai X., Gong J., Ye C., Xie L. (2019). Low-Grade Albuminuria Is Associated with Left Ventricular Hypertrophy and Diastolic Dysfunction in Patients with Hypertension. Kidney Blood Press. Res..

[29] Abdellatif Y., Nazmy N.M., Bastawy I., Raafat S. (2021). A Subtle Decline in Cardiac Mechanics is correlated with Albuminuria in Asymptomatic Normotensive Patients with Type 2 Diabetes Mellitus: A Two Dimensional Strain Echocardiography Study. J. Cardiovasc. Echogr..

[30] Landler N.E., Olsen F.J., Christensen J., Bro S., Feldt-Rasmussen B., Hansen D., Kamper A.-L., Christoffersen C., Ballegaard E.L.F., Sørensen I.M.H. (2022). Associations Between Albuminuria, Estimated GFR and Cardiac Phenotype in a Cohort with Chronic Kidney Disease: The CPH-CKD ECHO Study. J. Card. Fail..

[31] Liu M., He A., Wang Y., Chen C., Zhao X., Zhang S., Liang J., Hua M., Fang Z. (2020). Association of urine albumin-to-creatinine ratio with subclinical systolic dysfunction in hypertensive patients but not normotensive subjects: Danyang study. J. Clin. Hypertens..

[32] Jørgensen P.G., Biering-Sørensen T., Mogelvang R., Fritz-Hansen T., Vilsbøll T., Rossing P., Jensen J.S. (2018). Presence of micro- and macroalbuminuria and the association with cardiac mechanics in patients with type 2 diabetes. Eur. Heart J.-Cardiovasc. Imaging.

[33] Patro P.K., Dash B.K., Choudhury S., Sethy R.C. (2021). Study of Microalbuminuria in Type 2 Diabetes Mellitus as a Predictor of Left Ventricular Dysfunction-A Cohort Study. J. Clin. Diagn. Res..

[34] Husain-Syed F., DiFrancesco M.F., Deo R., Barr R.G., Scialla J.J., A Bluemke D., A Kronmal R., Lima J.A.C., Praestgaard A., Tracy R.P. (2023). Associations between eGFR and albuminuria with right ventricular measures: The MESA-Right Ventricle study. Clin. Kidney J..

[35] Boorsma E.M., ter Maaten J.M., Damman K., van Essen B.J., Zannad F., van Veldhuisen D.J., Samani N.J., Dickstein K., Metra M., Filippatos G. (2023). Albuminuria as a marker of systemic congestion in patients with heart failure. Eur. Heart J..

[36] Gori M., Senni M., Gupta D.K., Charytan D.M., Kraigher-Krainer E., Pieske B., Claggett B., Shah A.M., Santos A.B.S., Zile M.R. (2014). Association between renal function and cardiovascular structure and function in heart failure with preserved ejection fraction. Eur. Heart J..

[37] Wang Y., Zhao X., Zhai M., Fan C., Huang Y., Zhou Q., Tian P., An T., Zhang Y., Zhang J. (2021). Elevated urinary albumin concentration predicts worse clinical outcomes in hospitalized acute decompensated heart failure patients. ESC Heart Fail..

[38] Tao J., Sang D., Zhen L., Zhang X., Li Y., Wang G., Chen S., Wu S., Zhang W. (2023). Elevated urine albumin-to-creatinine ratio increases the risk of new-onset heart failure in patients with type 2 diabetes. Cardiovasc. Diabetol..

[39] Sharma A., Inzucchi S.E., Testani J.M., Ofstad A.P., Fitchett D., Mattheus M., Verma S., Zannad F., Wanner C., Kraus B.J. (2023). Kidney and heart failure events are bidirectionally associated in patients with type 2 diabetes and cardiovascular disease. ESC Heart Fail..

[40] Alataş Ö.D., Biteker M., Demir A., Yıldırım B., Acar E., Gökçek K., Gökçek A. (2022). Microalbuminuria and its Prognostic Significance in Patients with Acute Heart Failure with Preserved, Mid-Range, and Reduced Ejection Fraction; [Microalbuminúria e seu Significado Prognóstico em Pacientes com Insuficiência Cardíaca Aguda com Fração de Eje]. Arq. Bras. Cardiol..

[41] Barzilay J.I., Farag Y.M.K., Durthaler J. (2024). Albuminuria: An Underappreciated Risk Factor for Cardiovascular Disease. J. Am. Heart Assoc..

[42] Claudel S.E., Waikar S.S., Schmidt I.M., Vasan R.S., Verma A. (2023). The relationship between low levels of albuminuria and cardiovascular mortality among apparently healthy adults. medRxiv.

[43] Ioannou A., Rauf M.U., Patel R.K., Razvi Y., Porcari A., Martinez-Naharro A., Venneri L., Bandera F., Virsinskaite R., Kotecha T. (2024). Albuminuria in transthyretin cardiac amyloidosis: Prevalence, progression and prognostic importance. Eur. J. Heart Fail..

[44] Jørgensen P.G., Jensen M.T., Mogelvang R., von Scholten B.J., Bech J., Fritz-Hansen T., Galatius S., Biering-Sørensen T., Andersen H.U., Vilsbøll T. (2016). Abnormal echocardiography in patients with type 2 diabetes and relation to symptoms and clinical characteristics. Diabetes Vasc. Dis. Res..

[45] Edmonston D.L., Parikh K.S., Rajagopal S., Shaw L.K., Abraham D., Grabner A., Sparks M.A., Wolf M. (2020). Pulmonary Hypertension Subtypes and Mortality in CKD. Am. J. Kidney Dis. Off. J. Natl. Kidney Found..

[46] Wang L., Zhang W., Zhang C., Yan Z., Li S., Zhang C., Chen Y., Pan Q., Liang X., Chen X. (2022). Prognostic effect of pulmonary hypertension in patients with chronic kidney disease: Univariate and multivariate analyses of factors associated with survival. Front. Med..

[47] Reque J., Garcia-Prieto A., Linares T., Vega A., Abad S., Panizo N., Quiroga B., Boira E.J.C., López-Gómez J.M. (2017). Pulmonary Hypertension Is Associated with Mortality and Cardiovascular Events in Chronic Kidney Disease Patients. Am. J. Nephrol..

[48] Park M., Hsu C.-Y., Li Y., Mishra R.K., Keane M., Rosas S.E., Dries D., Xie D., Chen J., He J. (2012). Associations between kidney function and subclinical cardiac abnormalities in CKD. J. Am. Soc. Nephrol..

