# Supplementary material for: Renal Biomarkers and Prognosis in HFpEF and HFrEF: The Role of Albuminuria and eGFR—A Systematic Review
Source: Medicina (Kaunas). 2025 Jul 30;61(8):1386. doi: 10.3390/medicina61081386 (PMC12387696; doi:10.3390/medicina61081386)
Supplement: Supplementary file 1 [file medicina-61-01386-s001.zip › Supplementary_Rationale,Objectives,Search strategy.pdf]

Renal Biomarkers and Prognosis in HFpEF and HFrEF: The Role of Albuminuria and eGFR—A Systematic Review

THE RATIONALE OF REVIEW

This review aims to evaluate the prognostic significance of albuminuria and eGFR in patients diagnosed with heart failure (HF), specifically comparing outcomes between HFpEF and HFrEF subgroups.

The rationale for conducting a systematic review on the prognostic significance of albuminuria and estimated glomerular filtration rate (eGFR) in patients with heart failure (HF), comparing those with preserved ejection fraction (HFpEF) and reduced ejection fraction (HFrEF), arises from the following considerations:

Clinical Importance: HF is a complex and debilitating condition associated with significant morbidity and mortality. Identifying prognostic markers that can help stratify risk and guide management decisions is crucial for optimizing patient outcomes.

Renal Dysfunction in HF: Chronic kidney disease (CKD), as evidenced by albuminuria and decreased GFR, is highly prevalent in patients with HF and is associated with worse outcomes. Understanding the prognostic significance of these renal biomarkers in HFpEF and HFrEF populations is essential for comprehensive risk assessment and management.

Differences Between HFpEF and HFrEF: HFpEF and HFrEF represent distinct phenotypes of HF with differing pathophysiological mechanisms, clinical profiles, and outcomes. Exploring whether albuminuria and GFR have differential prognostic value in these subgroups can provide insights into the underlying mechanisms and guide tailored management strategies.

Clinical Decision-Making: Incorporating prognostic markers such as albuminuria and/or GFR into risk prediction models may enhance risk stratification and inform treatment decisions in patients with HF. Understanding the comparative prognostic value of these markers in HFpEF versus HFrEF can help clinicians optimize patient care.

Evidence Gap: While individual studies have examined the prognostic significance of albuminuria and eGFR in HF, there is a need for a comprehensive synthesis of the existing literature to provide a clearer understanding of their predictive value across different HF phenotypes.

By systematically reviewing and synthesizing the available evidence, this review aims to address these gaps in knowledge, providing clinicians and researchers with valuable insights into the prognostic significance of albuminuria and eGFR in patients with HF, and their comparative utility in HFpEF versus HFrEF populations.

OBJECTIVES

The objectives of the systematic review on the prognostic significance of albuminuria and estimated glomerular filtration rate (eGFR) in patients with heart failure, comparing those with preserved ejection fraction (HFpEF) and reduced ejection fraction (HFrEF), are as follows:

To Evaluate Prognostic Significance: Assess the prognostic significance of albuminuria and eGFR as biomarkers in patients diagnosed with HF. This involves examining their association with cardiovascular events, mortality, hospitalizations, and other relevant outcomes.

To Compare HFpEF and HFrEF Populations: Compare the prognostic value of albuminuria and eGFR between patients with HFpEF and those with HFrEF. Identify any differences in the strength of association and predictive accuracy of these renal biomarkers across different heart failure phenotypes.

To Investigate Differential Outcomes: Explore whether albuminuria and eGFR have differential prognostic implications in terms of cardiovascular events, mortality rates, and other outcomes between HFpEF and HFrEF subgroups. This involves synthesizing data from studies that specifically stratify outcomes based on ejection fraction status.

To Assess Clinical Utility: Evaluate the clinical utility of albuminuria and eGFR in risk prediction, patient stratification, and treatment decision-making in the management of heart failure. Determine whether incorporating these renal biomarkers into risk assessment models improves risk stratification and guides personalized treatment approaches.

To Identify Research Gaps: Identify gaps in the existing literature and highlight areas for future research. This includes identifying inconsistencies or limitations in the evidence base, as well as areas where further investigation is warranted to better understand the prognostic implications of albuminuria and eGFR in heart failure.

Overall, the objectives of this review are to provide a comprehensive synthesis of the available evidence on the prognostic significance of albuminuria and eGFR in patients with HF, compare outcomes between HFpEF and HFrEF populations, and assess their clinical utility in guiding patient management.

SEARCH STRATEGY

PUB-MED

(("Heart Failure, Diastolic"[Mesh] OR "Heart Failure, Systolic"[Mesh] OR "Cardiac Output, Low"[Mesh] OR "Diastolic Dysfunction"[Mesh] OR "Ejection Fraction"[Mesh] OR "Heart Failure"[Mesh]) OR ("Heart Failure, Diastolic" OR "Heart Failure, Systolic" OR "Cardiac Output, Low" OR "Diastolic Dysfunction" OR "Ejection Fraction" OR "Heart Failure")) AND (("Albuminuria"[Mesh] OR "Glomerular Filtration Rate"[Mesh] OR "Creatinine"[Mesh]) OR ("Albuminuria" OR "Glomerular Filtration Rate" OR "GFR" OR "Creatinine" OR "chronic kidney disease" OR "CKD" OR "renal insuficiency"))

SCOPUS LIBRARY

(TITLE-ABS-KEY ((("Heart Failure, Diastolic" OR "Heart Failure, Systolic" OR "Cardiac Output, Low" OR "Diastolic Dysfunction" OR "Ejection Fraction" OR "Heart Failure") OR ("Heart Failure, Diastolic" OR "Heart Failure, Systolic" OR "Cardiac Output, Low" OR "Diastolic Dysfunction" OR "Ejection Fraction" OR "Heart Failure")) AND (("Albuminuria" OR "Glomerular Filtration Rate" OR "GFR" OR "Creatinine") OR ("Albuminuria" OR "Glomerular Filtration Rate" OR "GFR" OR "Creatinine" OR "chronic kidney disease" OR "CKD"))) AND PUBYEAR > 2013 AND PUBYEAR < 2025) AND (albuminuria) AND (heart AND failure) AND (LIMIT-TO (OA , "all")) AND (LIMIT-TO (DOCTYPE , "ar")) AND (LIMIT-TO (LANGUAGE , "English"))

WEB OF SCIENCE

(TS=("heart failure" OR "diastolic heart failure" OR "systolic heart failure" OR "low cardiac output" OR "diastolic dysfunction" OR "ejection fraction") AND TS=("albuminuria" OR "glomerular filtration rate" OR "GFR" OR "creatinine"))
