# Supplementary material for: Renal Biomarkers and Prognosis in HFpEF and HFrEF: The Role of Albuminuria and eGFR—A Systematic Review
Source: Medicina (Kaunas). 2025 Jul 30;61(8):1386. doi: 10.3390/medicina61081386 (PMC12387696; doi:10.3390/medicina61081386)
Supplement: Supplementary file 1 [file medicina-61-01386-s001.zip › Supplementary_Table_5 - NOS.pdf]

Newcastle-Ottawa Scale (NOS) Quality Assessment

Study (Author, Year)	Selection (0–4)	Comparability (0–2)	Outcome/Exposure (0–3)	Total Score (0–9)
LIU (2018)	4	2	3	9
AIUMTRAKUL (2021)	3	2	3	8
ARNLOV and NOWAK (2022)	4	2	3	9
SUNG (2016)	4	2	3	9
SEO (2017)	3	2	3	8
HANNA (2017)	3	2	3	8
FENG (2017)	3	2	3	8
JORGENSEN (2018)	3	2	3	8
LIU (2020)	3	2	3	8
ABDEL-LATIF (2021)	3	2	3	8
SYED (2023)	4	2	3	9
LANDLER (2022)	3	2	3	8
WANG (2019)	3	2	3	8
PATRO (2021)	3	2	3	8
KATZ (2014)	4	2	3	9
GORI (2014)	4	2	3	9
BOORSMA (2023)	4	2	3	9
ALATAȘ (2022)	3	2	3	8
WANG (2021)	3	2	3	8
TAO (2023)	4	2	3	9
SHARMA (2023)	4	2	3	9
