# Supplementary material for: Renal Biomarkers and Prognosis in HFpEF and HFrEF: The Role of Albuminuria and eGFR—A Systematic Review
Source: Medicina (Kaunas). 2025 Jul 30;61(8):1386. doi: 10.3390/medicina61081386 (PMC12387696; doi:10.3390/medicina61081386)
Supplement: Supplementary file 1 [file medicina-61-01386-s001.zip › Supplementary_Table_6 - GRADE.pdf]

GRADE Summary of Findings Table

Outcome	No. of studies (participants)	Effect estimate	Certainty (GRADE)	Risk of bias	Inconsistency	Indirectness	Imprecision	Publication bias	Overall judgment
All-cause mortality	15 studies (~45,000 participants)	HR 1.8 (1.4–2.3) for high UACR; lower eGFR also predictive	Moderate	Serious (due to observational design)	Not serious (results generally consistent)	Not serious	Serious (wide CI in smaller studies)	Undetected	Downgraded for risk of bias and imprecision
Cardiovascular events	12 studies (~40,000 participants)	HR 1.5 (1.2–1.9) for albuminuria	Low	Serious (retrospective data and inconsistent adjustment)	Serious (heterogeneity in outcome definitions)	Not serious	Serious (limited sample in subgroups)	Possible (small studies with positive results)	Downgraded for risk of bias, inconsistency, and imprecision
HF-related hospitalizations	10 studies (~30,000 participants)	OR 2.0 (1.6–2.5) for reduced eGFR in HFrEF	Moderate	Moderate (mostly prospective, some unclear bias)	Not serious	Not serious	Not serious	Undetected	Downgraded for risk of bias
